# Factors Associated With Research Productivity Among Canadian Plastic Surgery Residents

**DOI:** 10.1177/22925503261477392

**Published:** 2026-08-26

**Authors:** Colm Guyn, Justin Lee, Curtis Budden

**Affiliations:** 1 Faculty of Medicine & Dentistry, University of Alberta, Edmonton, AB, Canada; 2 Division of Plastic Surgery, Department of Surgery, University of Alberta, Edmonton, AB, Canada

**Keywords:** research productivity, resident, reconstructive surgery, cross-sectional, mixed-methods, productivité en recherche, résident, chirurgie reconstructive, transversal, méthodes mixtes

## Abstract

**Introduction:**

Research productivity is a key marker of academic development in surgical training, yet considerable variability exists among residents. Understanding factors influencing scholarly output is critical for shaping program design and providing targeted support for academic development. This study aims to explore factors associated with research productivity among Canadian plastic surgery residents.

**Methods:**

A mixed methodology was utilized. First, a survey was distributed across Canadian plastic surgery programs to capture demographics, resident and program characteristics, and productivity metrics. Associations were examined using nonparametric tests given the non-normal distribution of data. Residents volunteered for follow-up interviews; interviews were transcribed verbatim, deidentified, and underwent thematic analysis to contextualize the associations found in the quantitative data.

**Results:**

Forty residents completed the survey (response rate 24%). Median (Q1-Q3) total publications were 8.0 (3.0-11.8), including 5.0 (3.0-7.5) preresidency and 1.5 (0.0-4.0) during residency. Greater preresidency publications, weekly hours dedicated to research, dedicated research during medical school, and completion of a MSc/PhD during residency had associations with increased productivity (P < .05). Interviews (n = 8) generated six themes spanning program architecture, time/capacity, infrastructure and administrative delays, supervisory engagement, baseline preparedness, and resident motivations/strategy.

**Conclusions:**

Resident research productivity is influenced by various interacting resident- and program-level factors. Our survey found that research productivity was associated with prior research experience, self-directed engagement, and research training through residency. Integrating these findings with our interviews we suggest programs may enhance productivity by strengthening early research onboarding, aligning expectations with usable protected time, improving coordination/analytic supports, and reducing administrative barriers.

## Résumé

**Introduction :** La productivité en recherche est un indicateur clé de la progression scientifique en formation chirurgicale, malgré une variabilité considérable entre résidents. Il est essentiel de comprendre les facteurs qui influent sur la production scientifique pour éclairer la conception des programmes et offrir un soutien ciblé à la progression scientifique. La présente étude vise à explorer les facteurs associés à la productivité en recherche des résidents canadiens en chirurgie plastique. **Méthodes :** Les chercheurs ont privilégié une méthodologie mixte. Ils ont d’abord distribué un sondage à l’ensemble des programmes canadiens de chirurgie plastique afin de recueillir des données démographiques, les caractéristiques des résidents et des programmes, ainsi que des indicateurs de productivité. Étant donné la distribution inhabituelle des données, ils ont examiné les associations à partir de tests non paramétriques. Des résidents se sont portés volontaires à des entretiens de suivi, lesquels ont été transcrits textuellement, dépersonnalisés, puis soumis à une analyse thématique afin de contextualiser les associations observées dans les données quantitatives. **Résultats :** Au total, 40 résidents ont rempli le sondage (taux de réponse de 24 %). Ils avaient publié un nombre médian (Q1 à Q3) de 8,0 publications (3,0 à 11,8), soit 5,0 avant la résidence (3,0 à 7,5) et 1,5 pendant la résidence (0,0 à 4,0). Un plus grand nombre de publications avant la résidence, des heures consacrées à la recherche chaque semaine, des périodes réservées à la recherche pendant les études médicales et l’obtention d’une maîtrise ou d’un doctorat pendant la résidence étaient associés à une plus grande productivité (p < 0,05). Les entretiens (n=8) ont permis de dégager six thèmes : l’architecture des programmes, le temps ou la capacité, l’infrastructure et les délais administratifs, l’investissement des superviseurs, la préparation de base et les motivations ou la stratégie des résidents. **Conclusions :** La productivité des résidents en recherche dépend de divers facteurs interreliés entre les résidents et le programme. Le sondage a révélé une association entre la productivité en recherche et l’expérience de recherche antérieure, l’engagement autonome et la formation en recherche acquise pendant la résidence. L’intégration de ces résultats à ceux des entretiens laisse supposer que, pour améliorer la productivité, les programmes pourraient renforcer l’intégration rapide à la recherche, faire concorder les attentes avec le temps protégé vraiment disponible, accroître le soutien à la coordination et à l’analyse et réduire les obstacles administratifs.

## Introduction

Research is an important component of residency within the evolving field of plastic and reconstructive surgery.^1^ For residents, research participation enhances their ability to critically evaluate literature and apply new knowledge to patient care.^2,3^ Research productivity is often used to appraise academic development during surgical training, influencing fellowship competitiveness and career opportunities.^4-6^ For residency programs, scholarly activity enhances institutional reputation, supports faculty advancement, and ultimately produces well-rounded, academically engaged surgeons.^7-9^ Nonetheless, residents seem to face substantial barriers to conducting research during residency, including clinical demands, administrative burden, and variable access to mentorship and resources.^3^

Despite these challenges, mechanisms shaping research productivity among Canadian plastic surgery residents remain incompletely understood. Prior work suggests resident research productivity is shaped by both resident- and program-level factors, including prior research experience, protected research time, mentorship access, and institutional supports.^3,9-19^ However, existing evidence is largely derived from U.S. institutions and is predominantly quantitative, limiting interpretation of the pathways by which these factors influence research productivity.

To address these gaps, we surveyed Canadian plastic surgery residents and conducted follow-up interviews with the aim of identifying resident- and program-level factors associated with research productivity, characterizing resident-described processes that enable or constrain research during training.

## Methods

### Study Design

We conducted a cross-sectional mixed-methods study using an explanatory sequential approach. Quantitative survey data identified resident-reported program and resident characteristics associated with productivity metrics. Follow-up qualitative interviews explored underlying mechanisms and contextual factors not captured by closed-ended survey items. Findings were then integrated using a joint display to identify convergence, complementarity, and discordance across datasets.^20,21^ Study procedures were approved by the University of Alberta Research Ethics Board (Pro00155760). All survey and interview participants provided informed consent.

### Recruitment

An anonymous online survey was distributed to all accredited plastic surgery residency programs in Canada (12 programs). Program directors forwarded the survey to residents, and a reminder was sent via the Canadian Society of Plastic Surgeons (CSPS) to registered residents. Volunteer interview participants were recruited from survey respondents, forming a convenience sample; maximum variation sampling was not used. All current Canadian plastic surgery residents were eligible (N = 165).

### Survey

#### Design

Survey development began with review of the literature on resident research productivity, mapping characteristics into resident- and program-level domains.^3,9,11^ An item-pool was generated, capturing demographics, research background, program expectations, supports, and productivity metrics. Plastic surgery faculty and residents reviewed content for relevance and completeness before a survey instrument was drafted and piloted with local residents. Feedback was used to improve wording, reduce ambiguity, and minimize response burden prior to distribution (Supplemental Table 1).

#### Analysis

Survey data were summarized using descriptive statistics. Categorical survey items were reported as frequencies and percentages. Research productivity was assessed using median (1) publication counts and (2) Hirsch index (h-index), a citation-based metric.^22,23^ Associations between self-reported resident/program characteristics and research productivity were calculated using non-parametric tests (Mann–Whitney U, Kruskal–Wallis, Spearman's correlation). Statistical analyses were performed using SPSS Statistics 290.1.0 (IBM Corp, Armonk, New York, USA). A P value less than .05 indicated significance.

### Interview

#### Interview Design and Collection

Interview design began with a targeted review of qualitative studies on resident research productivity. A semistructured interview guide was developed to explore resident accounts of residency research experiences, preresidency research exposure, program influences (eg, culture, mentorship, supports), and perceived barriers/facilitators to project progression, with probes for elaboration (Supplemental Table 2).^3^

Interviews were conducted via Zoom (Zoom Video Communications, San Jose, CA, USA) by a medical student with no supervisory/evaluative relationship with participants. Interviewing principles were reviewed by the study team and the guide was piloted with local residents and faculty giving feedback to support consistent questioning while preserving flexibility to probe emergent topics. Interviews were audio-recorded, transcribed verbatim, and deidentified before analysis.

Given the focused aim, specific participant group, literature- and survey-informed interview guide, and reflexive thematic analysis, eight interviews were considered sufficient.^20^ Later interviews reinforced existing themes without generating new domains. Formal thematic saturation was not used as a stopping criterion.

#### Thematic Analysis

Transcripts underwent reflexive thematic analysis to identify patterns in residents’ accounts and interpret how resident- and program-level factors were perceived to influence research productivity.^20^ De-identified transcripts were coded by a single analyst using a primarily deductive, semantic approach, whereby codes were applied to explicit meaning in the data. Given the focused interview sample, a single-coder was used; rigor was supported through repeated familiarization, line-by-line coding, cross-transcript comparison, and documentation of code definitions and theme development in a codebook/audit trail. Related codes were grouped into candidate themes and refined with the research team to ensure coherence, distinctiveness, and fit before finalization.

Quantitative and qualitative findings were integrated during interpretation by comparing survey associations with corresponding themes and representative quotations to identify convergence, complementarity, discordance, and potential explanatory mechanisms of productivity.

## Results

Of 165 eligible residents, 40 completed the survey. All completed surveys were included in analysis. Respondents represented 11 of 12 Canadian plastic surgery residency programs, all programs were included in the distribution process. Due to item nonresponse, sample sizes differed across variables and analyses.

### Survey Participant Demographics

Presented in Table 1, of the 40 survey respondents, 62.5% identified as male and 37.5% as female. Most respondents were 31 years or greater (47.5%), followed by 26 to 30 (35.0%) and 20 to 25 (17.5%). Relationship status was reported as single by 40.0%, living with a partner by 25.0%, and married by 35.0%. Few respondents reported dependents (15.0%). A Bachelor of Science was the most common undergraduate background (57.5%), while 12.5% had not completed an undergraduate degree. Postgraduate training before residency was reported by 40.0%. No significant associations were observed between demographics and research productivity metrics.

**Table 1.** Resident Characteristics and Productivity Metrics as Reported by Residents.

Variable	All N (%)	Publications in Residency Median (Q1-Q3)	*P* Value	Total Publications Median (Q1-Q3)	*P* Value	h-Index Median (Q1-Q3)	*P* Value
Age	40 (100%)		.345*^†^*		.883*^†^*		.773*
20–25	7 (17.5%)	0.0 (0.0-2.0)		8.0 (4.0-11.0)		3.0 (2.0-4.0)	
26–30	14 (35.0%)	2.0 (0.0-6.5)		9.0 (3.0-12.0)		3.5 (1.8-5.3)	
31+	19 (47.5%)	1.0 (0.0-5.0)		6.0 (3.0-12.0)		4.0 (1.3-6.8)	
Sex	40 (100%)		.489*		.619*		.927*
Female	25 (62.5%)	1.0 (0.0-2.0)		8.0 (5.0-11.0)		3.0 (2.0-5.3)	
Male	15 (37.5%)	2.0 (0.0-6.0)		8.0 (3.0-12.0)		4.0 (1.5-5.5)	
Relationship status	40 (100%)		.504*^†^*		.288*^†^*		.810*^†^*
Single	16 (40.0%)	1.5 (0.0-2.8)		10.0 (5.3-12.8)		3.0 (2.5-5.5)	
Partner	10 (25.0%)	1.0 (0.0-4.3)		5.5 (3.0-10.0)		4.0 (1.0-5.0)	
Married	14 (35.0%)	1.5 (0.0-8.8)		5.0 (2.8-14.0)		2.0 (1.0-6.0)	
Dependents	40 (100%)		.493*		.566*		.348*
No	34 (85.0%)	1.5 (0.0-4.0)		8.5 (3.0-11.3)		3.5 (2.0-4.3)	
Yes	6 (15.0%)	2.0 (0.0-15.0)		3.5 (1.8-20.0)		6.0 (—)	
Undergraduate	40 (100%)		.902*^†^*		.957*^†^*		.204*^†^*
BSc/HSc	29 (72.5%)	1.0 (0.0-4.5)		8.0 (3.0-11.5)		4.0 (2.0-6.0)	
Other Bach	3 (7.5%)	1.0 (0.0-1.0)		6.0 (3.0-6.0)		2.0 (1.0-2.0)	
CEGEP	3 (7.5%)	0.0 (0.0-0.0)		5.0 (0.0-5.0)		4.0 (0.0-4.0)	
DNC	5 (12.5%)	2.0 (1.0-8.5)		11.0 (1.5-15.0)		2.0 (1.0-2.0)	
PGY	40 (100%)		<.01*		.110*		.023*
1–2	14 (35.0%)	0.0 (0.0-1.3)		5.5 (2.8-10.0)		2.0 (1.5-3.5)	
3+	26 (65.0%)	2.5 (1.0-6.0)		10.0 (3.0-12.3)		4.0 (2.8-6.3)	
Prior postgrad	40 (100%)		.362*^†^*		.178*^†^*		.054*^†^*
No	24 (60.0%)	1.0 (0.0-2.0)		5.5 (3.0-10.8)		2.5 (1.3-4.0)	
Thesis based	12 (30.0%)	2.5 (0.3-6.0)		10.5 (6.5-12.0)		4.0 (2.5-6.0)	
Course based	4 (10.0%)	2.0 (0.3-3.8)		7.0 (1.8-19.0)		11.5 (6.0-11.5)	
Dedicated research time intra/post med	40 (100%)		.050*		.150*		.348*
No	39 (97.5%)	1.0 (0.0-4.0)		8.0 (3.0-11.0)		3.5 (2.0-4.3)	
Yes	1 (2.5%)	15.0 (—)		20.0 (—)		-	
CIP/MSc/PhD in residency	40 (100%)		.048*		.055*		.139*
No	36 (90.0%)	1.0 (0.0-3.0)		6.0 (3.0-11.0)		3.0 (2.0-4.0)	
Yes	4 (10.0%)	4.5 (2.5-9.5)		11.5 (10.0-22.8)		5.0 (3.3-8.3)	
Research block	36 (100%)		.919*^†^*		.398*^†^*		.763*^†^*
No	31 (86.1%)	2.0 (0.0-4.0)		6.0 (3.0-12.0)		4.0 (1.5-5.5)	
Completed	3 (8.3%)	3.0 (0.0-3.0)		10.5 (10.0-10.5)		4.0 (3.0-4.0)	
Planned	2 (5.6%)	2.0 (0.0-2.0)		11.0 (5.0-11.0)		-	
Fellowship	37 (100%)		.865*		.865*		.476*
No	1 (2.7%)	1.0 (—)		6.0 (—)		-	
Yes	36 (97.3%)	2.0 (0.0-4.8)		8.5 (3.0-12.0)		4.0 (2.3-5.8)	
Academic career	40 (100%)		.406*		.075*		.213*
No	10 (25.0%)	1.0 (0.0-2.3)		4.5 (2.8-7.3)		3.0 (1.8-4.0)	
Yes	21 (52.5%)	2.0 (0.0-6.0)		10.0 (5.5-13.0)		4.5 (3.0-6.8)	
Unsure	9 (22.5%)	0.0 (0.0-4.5)		4.0 (2.0-10.0)		2.0 (1.5-4.0)	
Hours per week for research	38 (100%)		.039^†^		.013^†^		.238*^†^*
0	4 (10.5%)	0.0 (0.0-0.0)		2.0 (0.3-3.0)		-	
1–2	20 (52.6%)	1.0 (0.0-2.8)		6.0 (3.3-10.0)		4.0 (1.5-4.0)	
3–4	6 (15.8%)	2.5 (0.0-8.3)		10.0 (3.0-14.0)		2.5 (1.3-5.3)	
5+	8 (21.1%)	2.5 (2.0-7.5)		12.0 (6.5-14.5)		4.5 (3.0-7.5)	
External funding	40 (100%)		.045^†^		.102^†^		.199^†^
No	28 (70.0%)	0.5 (0.0-2.8)		5.0 (3.0-10.8)		3.0 (1.0-6.0)	
Declined	6 (15.0%)	2.0 (1.5-6.5)		9.0 (5.0-12.3)		4.0 (4.0-4.8)	
Approved	6 (15.0%)	4.0 (1.8-7.3)		10.5 (7.5-17.8)		5.5 (2.5-8.5)	

*Note.* Characteristics reported as n (%) with associated productivity metrics reported as median (Q1 − Q3). PGY, postgraduate year; CIP, Clinician Investigator Program. *P* < .05 taken as significant. h-index n = 23. Resident characteristics collected form survey reported as n (%) with associated productivity metrics reported as median (Q3-Q1). *P* < .05 taken as significant. h-index n = 23.

* Calculated using Mann–Whitney U test. † Calculated using Kruskal–Wallis test.

### Resident Characteristics

Characteristics from Table 1 show, among all survey respondents (N = 40), 35.0% were junior residents (PGY1-2) and 65.0% were senior residents (PGY3+). Dedicated research during medical school was reported by 2.5%, and 10.0% reported completing graduate or clinician-investigator program (CIP) research training during residency, defined as CIP, MSc, or PhD in the survey. Academic career interest was reported by 52.5%, with 22.5% unsure and 25.0% not anticipating academic involvement. External funding was applied for by 30.0% of respondents, half of whom received funding. Among item respondents, most reported no planned research block (86.1%, N = 36), nearly all anticipated fellowship training (97.3%, N = 37), and weekly research time was most commonly 1–2 h (52.6%, N = 38), followed by 5 or more hours (21.1%), 3–4 h (15.8%), and 0 h (10.5%).

Across metrics of productivity, respondents reported a median (Q1-Q3) of 5.0 (3.0-7.50) preresidency publications, 1.5 (0.0-4.0) in-residency publications, and 8.0 (3.0-11.8) total publications. Median h-index was 4.0 (2.0-5.0) among respondents reporting this metric (N = 23). Preresidency publications correlated with in-residency publications (ρ = 0.321, *P* = .043), total publications (ρ = 0.836, *P* < .001), and h-index (ρ = 0.682, *P* < .001). Senior resident status was associated with higher in-residency publications (*P* < .001) and h-index (*P* = .023). Weekly time dedicated to research was associated with in-residency (H(3) = 8.34, *P* = .039) and total publications (H(3) = 10.79, *P* = .013), with posthoc differences only between residents reporting greater than five hours/week and zero hours/week after Bonferroni correction. Dedicated research during medical school (*P* = .050) and postgraduate training during residency (*P* = .048) were also associated with higher in-residency publication counts.

### Program Characteristics

Most respondents reported a formal research requirement (95.0%, N = 40) (Table 2). Among respondents to program-support items (N = 36), 66.7% reported protected research time, 66.7% reported a divisional research director, and 50.0% reported access to a designated research supervisor. Research meetings were most commonly quarterly (55.6%), followed by monthly (19.4%) or less frequent intervals (25.0%). Fewer residents reported access to research assistants/coordinators (19.4%), biostatisticians (25.0%), IT/data support (5.6%), or internal funding (11.1%). No significant associations were observed between resident-reported program characteristics and research productivity metrics.

**Table 2.** Program Characteristics and Productivity Metrics as Reported by Residents.

Variable	All N (%)	Publication in Residency Median (Q1-Q3)	*P* Value	Total Publications Median (Q1-Q3)	*P* Value	h-Index Median (Q1-Q3)	*P* Value
Research requirement	39 (100.0%)		.297*		.097*		.909*
No	2 (5.1%)	4.5 (2.0-7.0)		13.0 (13.0-13.0)		-	
Yes	37 (94.9%)	1.0 (1.0-4.0)		6.0 (3.0-11.0)		4.0 (2.0-6.0)	
Protected time	36 (100.0%)		.476*		.608*		.157*
No	12 (33.3%)	1.0 (0.0-3.8)		6.0 (3.0-11.5)		5.0 (2.0-9.5)	
Yes	24 (66.7%)	2.0 (0.0-6.0)		8.5 (3.25-12.0)		3.0 (1.0-5.0)	
Research Meetings	36 (100.0%)		.808*^†^*		.242*^†^*		.580*^†^*
Monthly	7 (19.4%)	2.0 (0.0-11.0)		6.0 (3.0-20.0)		3.5 (2.8-7.3)	
Quarterly	20 (55.6%)	2.0 (0.0-3.8)		7.0 (3.0-11.0)		2.0 (1.0-6.0)	
Less than quarterly	9 (25.0%)	2.0 (0.5-6.5)		10.0 (5.5-16.5)		5.0 (1.0-8.3)	
Research director	36 (100.0%)		.361*		.166*		.808*
No	12 (33.3%)	2.0 (0.3-6.0)		12.0 (3.5-14.5)		4.5(1.5-6.8)	
Yes	24 (66.7%)	1.0 (0.0-4.0)		7.0 (3.0-10.0)		3.0 (2.0-5.0)	
Research supervisor	36 (100.0%)		.606*		.501*		.972*
No	18 (50.0%)	1.5 (0.0-6.0)		6.0 (3.0-12.0)		4.0 (2.0-6.0)	
Yes	18 (50.0%)	1.5 (0.0-3.3)		9.5 (4.5-13.0)		3.5 (1.8-6.3)	
Research Assistant	36 (100.0%)		.325*		.306*		.471*
No	29 (80.6%)	1 .0(0.0-4.5)		8.0 (3.0-11.5)		4.0 (2.0-6.0)	
Yes	7 (19.4%)	2.0 (1.0-6.0)		10.0 (5.0-13.0)		2.0 (1.0-2.0)	
Biostatistician	36 (100.0%)		.830*		.312*		.011*
No	27 (75.0%)	1.0 (0.0-5.0)		10.0 (4.0-12.0)		4.0 (2.5-6.5)	
Yes	9 (25.0%)	2.0 (0.5-4.0)		3.0 (2.5-12.0)		1.5 (1.0-2.8)	
Data support	36 (100.0%)		.578*		.714*		.533*
No	34 (94.4%)	1.5 (0.5-5.3)		8.5 (3.0-12.0)		4.0 (2.0-6.0)	
Yes	2 (5.6%)	1 (0.0-1.0)		8.5 (4.0-8.5)		2.5 (2.0-2.5)	
Int funding	36 (100.0%)		.942*		.827*		.286*
No	32 (88.9%)	1.0 (0.0-5.8)		8.5 (3.0-12.0)		4.0 (2.0-6.0)	
Yes	4 (11.1%)	2.0 (0.5-3.5)		6.5 (3.0-12.3)		2.0 (1.0-2.0)	

*Note.* Characteristics reported as n (%) with associated productivity metrics reported as median (Q3-Q1). *P* < .05 taken as significant. h-index n = 23. Program characteristics collected from survey reported as n (%) with associated productivity metrics reported as median (Q3-Q1). *P* < .05 taken as significant. h-index n = 23.

* Calculated using Mann–Whitney U test. † Calculated using Kruskal–Wallis test.

### Thematic Analysis

**Figure 1.**
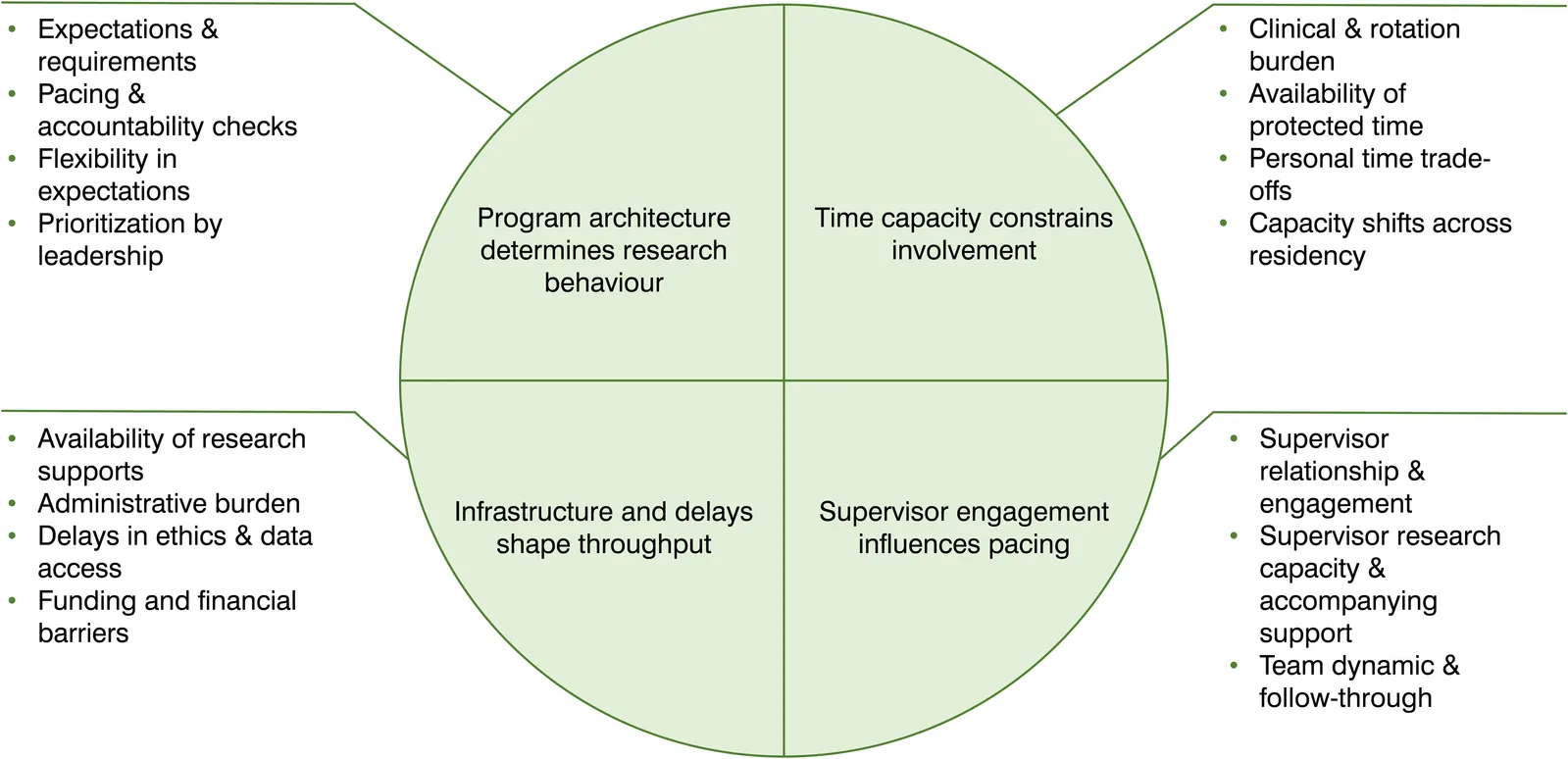
Program-Level Themes Identified in Resident Interviews (n = 8).

*Note.* Various codes are linked to each theme representing consolidated domains of meaning identified through iterative coding during thematic analysis.

**Figure 2.**
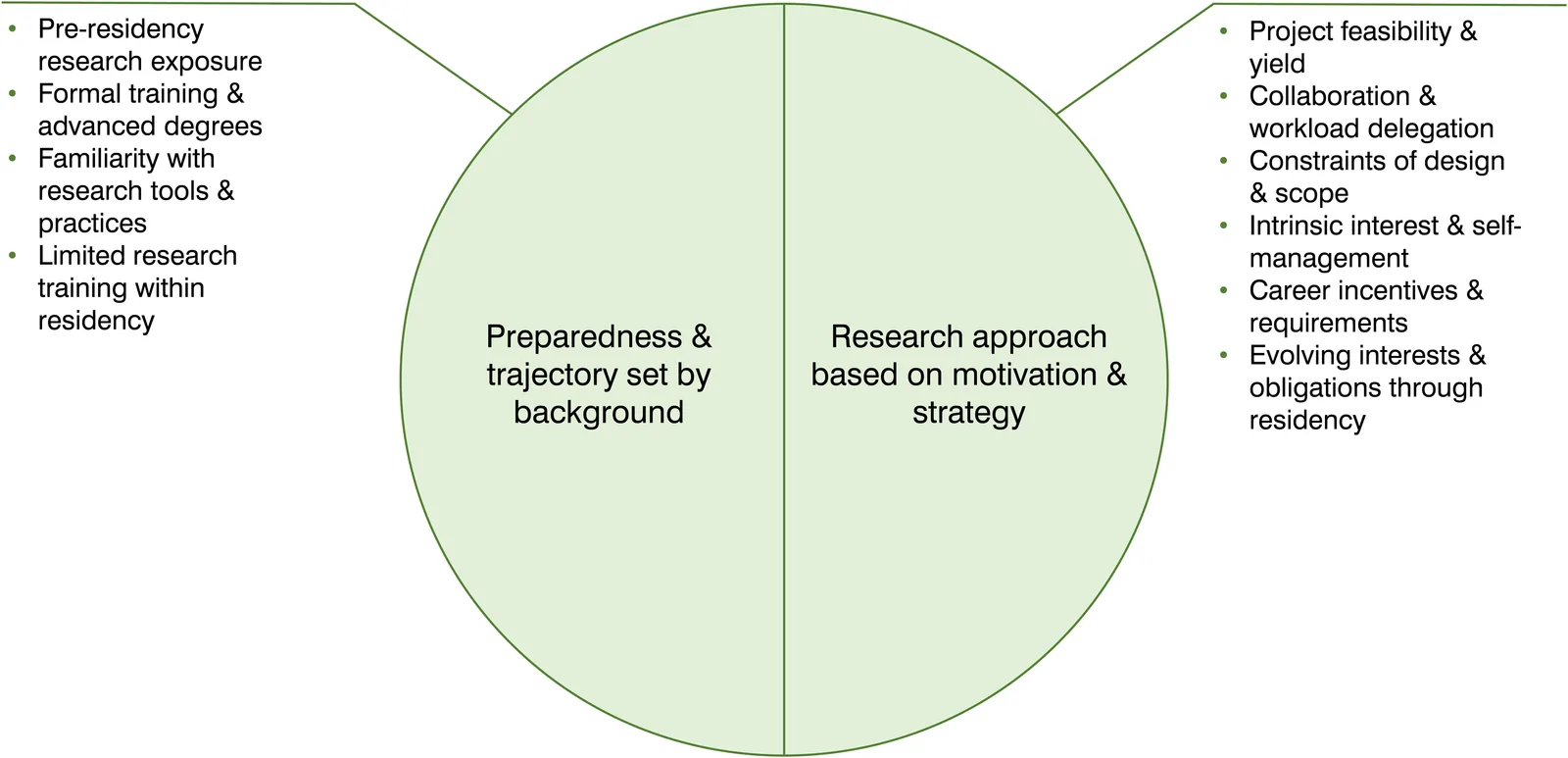
Resident Specific Themes Identified in Resident Interviews (n = 8).

*Note.* Various codes are linked to each theme representing consolidated domains of meaning identified through iterative coding during thematic analysis.

Interviews (N = 8) included participants from 5 residency programs, with equal representation of males and females. Participants included 6 senior and 2 junior residents; 5 expressed academic career interest, 2 were unsure, and 1 was not interested. Analysis generated 6 distinct themes influencing research productivity, grouped into 4 system-level themes shaping research conditions (Figure 1) and 2 resident-level themes describing how residents navigate these conditions (Figure 2). Six themes were retained because each captured a distinct mechanism shaping productivity.

#### Theme 1: Program Architecture Determines Research Behavior

Residents described program architecture as shaping research behavior through requirements, checkpoints, expectations, and accountability. Structured programs supported earlier project initiation and follow-through, while less structured environments depended more on resident self-direction. As one participant noted, built-in meetings to “go over a project [and] see what needs to be done … helps.” In contrast, limited expectations could deprioritize project completion: “Not much expectation to publish … not a priority.”

#### Theme 2: Time and Capacity Constraints

Residents described clinical responsibilities as limiting both the time and energy for research, often pushing scholarly work into evenings, weekends, or gaps between clinical duties. As one resident explained, “working so much, it's difficult to find time, but also energy.” Participants emphasized that research productivity was most useful when substantial, continuous, and aligned with project timelines; one resident identified “two months of dedicated research” as “the biggest help.” Shorter or poorly timed blocks were viewed as less efficient because they lacked sufficient continuity to advance projects meaningfully.

#### Theme 3. Infrastructure and Delays Shape Throughput

Residents described infrastructure as shaping productivity by affecting research momentum, burden, and the effort needed to navigate institutional processes. Research coordinators, statisticians, and funding reduced administrative and technical burdens; one resident noted that “hiring a statistician was very helpful.” In contrast, ethics approvals, data access, and other administrative requirements created friction and delays, with one resident describing “ethics and data access” as the “biggest bottlenecks” after a project took “seven months to [access available] data.”

#### Theme 4. Supervisor Engagement Influences Pacing

Supervisors were described as shaping project pace by influencing scope, resource access, and progression toward completion. Residents emphasized that engagement and responsiveness mattered more than simply having an assigned supervisor. Supportive supervisors helped refine feasible projects, navigate barriers, maintain momentum, and connect residents with resources. As one participant noted, “[You] can only do as much as the PI is willing to support.” Conversely, delayed feedback, limited availability, or poor follow-through caused projects to stall despite resident effort.

#### Theme 5. Preparedness and Trajectory Set by Background

Residents described prior research exposure and formal training as improving preparedness through familiarity with ethics, methodology, analysis, and project management. This enabled faster initiation, greater independence, and more realistic planning. Conversely, limited experience made project initiation more difficult, as “some residents are hesitant to start because they don't know how.” Residents noted that limited research-skills teaching during residency may allow baseline differences to persist, widening productivity gaps.

#### Theme 6. Research Approach Based on Motivation and Strategy

Residents described motivation and strategic decision-making as influencing whether limited research opportunities translated into scholarly output. Motivation was shaped by program requirements, fellowship or academic goals, and intrinsic interest, but alone could not sustain productivity during residency. Residents adapted with feasible project selection, resident collaboration, task delegation and recalibrating involvement as interests, clinical responsibilities, and personal obligations changed. As one participant described, this involved asking “what can I meaningfully do in a succinct amount of time and get published.”

### Integrated Findings

Integrated findings are summarized in Table 3. Survey findings were compared with qualitative themes to identify convergence, complementarity, and discordance. Associations with weekly research time, graduate or clinician–investigator training during residency, and prior research experience converged with themes emphasizing sustained time, preparedness, and procedural fluency. Conversely, several resident-reported program supports were not associated with productivity despite being described as important for research throughput, possibly reflecting a perceived reduction in burden without directly increasing publication volume.

**Table 3.** Joint Display Integrating Survey Findings and Qualitative Themes.

Qualitative Theme	Representative Quotation	Survey Item	*P* Value	Integrated Interpretation
Program architecture determines research behavior	“Not much expectation to publish … it's not much of a priority”	Research requirement Research meetings Research director	.297 .808 .361	Although reported measures of program structure were not significantly associated with productivity, residents described structures as improving productivity when expectations, checkpoints, and accountability were cooperatively enforced. This suggests that structure implementation may be more important than the simple presence alone, which is difficult to capture with binary survey measures. Alternatively, the overall effect on productivity may be modest compared to perceived benefit.
	“Meetings where we sit down, go over a project, see what needs to be done … that structure built into the program helps.”			
	“Consistent pressure to produce … increases productivity compared to programs with looser requirements.”			
Time and capacity constraints	“Working so much, it's difficult to find time, but also energy”	Weekly research time Protected time Research block CIP/MSc/PhD training during residency	**.039** .476 .919 **.048**	Survey and interview findings converged around the importance of sustained research time. Interviews suggested that the quality and continuity of research time may be more important than nominal protected time alone.
	“Two months of dedicated research time, for sure, the biggest help.”			
	“We don't have dedicated research blocks, even one month of protected time would make a huge difference”			
Infrastructure and delays shape throughput	“Hiring a statistician was very helpful”	Research Assistant Biostatistician Data support Internal funding	.325 .830 .578 .942	Resident interviews emphasized how well implemented/accessible supports reduced administrative and analytic burden. However, no association was based on resident survey reports of support availability. This may once again reflect the perceived reduction in research burden without causing a significant change in research volume, while also acknowledging survey limitations in capturing differences in implementation.
	“I think not having a research coordinator is tough”			
	“People who help with submissions for the ethics board … actually helped a lot”			
	“Biggest bottlenecks are ethics and data access … seven months to obtain data”			
Supervisor engagement influences pacing	“Staff who are invested [is important] … can only do as much as the PI is willing to support.”	Research supervisor	.606	Survey-reported access to a research supervisor was not significantly associated with productivity, but interviews suggested that the quality of supervision was more important than nominal access. Residents described supervisor responsiveness, engagement, and ability to mobilize resources as key determinants project progression.
	“Stalling with one of the staff … didn't end up going through with publication.”			
	“Very supervisor-dependent … mine have RAs who help with ethics, grant applications, and statistics”			
Preparedness and trajectory set by background	“Some residents are hesitant to start because they don't know how.”	Preresidency publications Dedicated research during medical school Prior postgraduate degree	**.043** **.050** .362	Survey and interview findings converged in suggesting that prior research experience may support productivity through greater confidence, procedural fluency, and independence. The lack of association with prior postgraduate degree may reflect that degree status is a less precise marker of publishable research experience and may involve research contexts that are less directly transferable to residency research.
	“We don't get formal teaching on writing ethics or grant proposals …”			
	“Understanding what research looks like … research processes and ethics … connections from my master's.”			
Research approach based on motivation and strategy	“Motivation driven by requirements … fellowship … an academic career”	Weekly research time Fellowship Academic career interest Program requirements	**.039** .865 .406 .297	Survey findings found that productivity was more closely associated with weekly research time than stated career intentions alone. Interviews explained this by showing how motivation may operate indirectly through behaviors: residents translated interest, fellowship goals, and program expectations into output through sustained engagement, feasible project selection, collaboration, and delegation. This suggests that motivation influences productivity less as a static characteristic and more through the strategies residents use to convert limited time into completed projects.
	“I have a genuine interest in research …”			
	“Take something sizeable … what can I do in a succinct amount of time and get published”			
	“[Residents] working on multiple projects, and there's a lot of collaboration because everyone is trying to be productive.”			

*P* values are taken from survey item correlations with in-residency publication counts. *P* < .05 taken as significant (bolded).

## Discussion

Research is a valuable component of surgical training, yet variation in resident involvement is incompletely explained by quantitative metrics alone.^6,9-19^ This national mixed-methods study integrated survey and interview data to examine factors associated with research productivity in Canadian plastic surgery residency and explore resident-described mechanisms shaping output.

From survey analyses, self-reported in-residency productivity was associated with preresidency publications, dedicated research during medical school, weekly research time, and graduate research training during residency, while resident-reported program supports showed no significant associations. Summarized in the joint display (Table 3), qualitative findings contextualized observed associations and null findings by identifying perceived influences poorly captured by closed-ended survey items.

### Program Influences on Research Productivity

Resident-reported program characteristics were not significantly associated with productivity metrics, yet interviews suggested they shape the conditions needed to progress projects from conception to publication.

#### Program Architecture and Expectations

Resident-reported program structure, including research requirements, meetings, and directors were not significantly associated with productivity, contrasting prior literature suggesting structured expectations and curricula support scholarly output.^15-18^ However, interviews suggested program architecture influences research behavior when expectations, checkpoints, and accountability are clearly implemented and reinforced. The lack of quantitative association may reflect a perceived improvement in project initiation, momentum, and follow-through, without substantial change in publication-based productivity metrics. Additionally, with variability in implementation, binary survey capture may be a poor proxy for impact. Given research requirement prevalence, programs may further enhance resident research with improved implementation of early onboarding, staged milestones, clear expectations, and accountability.

#### Time Capacity as Constraint

Survey and interview findings converged in emphasizing research time and capacity as important for productivity. Weekly research time and completion of graduate or clinician-investigator training during residency were associated with in-residency publications, consistent with prior literature.^3,19^ These should be interpreted cautiously, weekly research time also reflects resident motivation and engagement, while graduate/CIP training reflects a bias for pre-existing research interests, mentorship access, and institutional support. Although protected time and research blocks were not associated with productivity, interviews suggested time was most useful when substantial, continuous, and aligned with project stage. Thus, the impact of research time may depend less on its presence alone and more on how it is structured, timed, and integrated within residents’ clinical and research contexts.

#### Infrastructure and Bureaucratic Delays

Resident-reported access to research assistants/coordinators, biostatistical support, IT/data support, and internal funding was not associated with productivity metrics, contrary to literature linking infrastructure to resident productivity.^3,10^ However, interviews characterized infrastructure and bureaucratic delays as central mechanisms shaping research productivity. Residents described administrative support, statistical assistance, and funding as reducing research burden, with ethics and data-access perceived as delaying progress. The discrepancy may again reflect an influence over research processes without directly increasing volume-based productivity metrics. Additionally, binary measures of support availability may not adequately capture resources accessibility, implementation, or frequency of use. Streamlining ethics and data-access pathways and improving access to practical supports may reduce resident burden, even if effects on publication counts are limited or difficult to detect.

#### Supervisors as Gatekeepers

Resident-reported access to research supervisors and directors was not associated with productivity metrics, contrasting studies linking mentorship with resident productivity.^3,4,12,15,16^ Interviews elaborate on the substance of supervision—engagement, responsiveness, and resources—shaping project momentum. Thus, absence of a survey association should not be interpreted as evidence supervision is unimportant; rather, access alone may incompletely capture supervisory contribution. Research throughput may be improved by clarifying supervisor-resident expectations at project outset and intentionally pairing residents with supervisors whose capacity and approach align with resident goals.

### Resident Influences on Research Productivity

#### Preparedness and Trajectory

Echoing prior studies, our quantitative results suggest productivity is associated with research experience, specifically preresidency publications and dedicated research during medical school.^9,11-14^ Prior postgraduate education was not associated with productivity, possibly reflecting heterogeneity in degree type, research intensity, and transferability to residency projects. Interviews suggested prior experience builds procedural fluency with ethics, approvals, methods, and project management, encouraging earlier start-up and independence. Conversely, residents lacking research backgrounds may struggle to initiate projects, while limited residency-based research training allows baseline differences to persist. Importantly, these findings do not support resident selection based on prior publications, which likely reflects opportunity and mentorship as much as individual ability. Rather, they identify a modifiable mechanism that may be addressed through early research onboarding and practical skills support.

#### Motivation and Strategy

In contrast to studies linking academic trajectory and structured requirements with scholarship, survey analysis showed sustained engagement (weekly research time) was associated with productivity, whereas explicit motivators (fellowship, academic career, program requirements) were not.^4,10,17^ Interviews suggested motivation may indirectly act on productivity through behaviors such as time investment, feasible project selection, and collaboration. Residents described adapting their research approach to clinical constraints by balancing interests and career goals with achievable projects. Thus, improving productivity may be most effective when programs help residents translate varying levels of motivation into productive research behaviors within supportive research environments offering usable time, mentorship, and support for project completion.

## Limitations

Key limitations relate to sampling, measurement, and qualitative scope. Modest survey response rate (24%) increases risk of nonresponse and selection bias, limits subgroup precision, and reduces confidence in program-level comparisons. Respondents represented 11 of 12 Canadian programs, limiting transferability across all programs. Program characteristics were resident-reported, analyzed at the respondent level, and not aggregated by program, limiting inference about program-level effects. Productivity measures were self-reported; publication counts may not reflect contribution, quality, or broader scholarship, and h-index reporting was incomplete. A small volunteer interview sample (n = 8) and subset of programs (n = 5) introduced volunteer bias and limits generalizability. Although information power guided sample adequacy, thematic saturation was not assessed, and additional interviews may have expanded under-represented perspectives. Finally, thematic analysis was conducted by a single coder; despite an iterative codebook and audit trail, themes remain interpretive and alternative interpretations are possible.^20^

## Conclusions

Resident research productivity is shaped by multiple individual and system-level factors, influencing project progress from conception to completion.^3,9-19^ Productivity was associated with preresidency publications, dedicated research during medical school, weekly research time, and graduate research training during residency. Interviews contextualized these associations by describing how program architecture, time constraints, support accessibility, supervisor relationships, resident preparedness, motivation, and strategy shape research momentum alongside clinical demands. Together, these findings suggest improving productivity requires attention not only to the presence of research supports, but to how they are implemented, accessed, and integrated in residency. Programs may benefit from strengthening early research onboarding, aligning expectations with usable protected time, improving practical resource access, clarifying supervisor-resident expectations, and supporting strategies that help residents balance research goals with clinical demands.

## Supplemental Material

sj-docx-1-psg-10.1177_22925503261477392 - Supplemental material for Factors Associated With Research Productivity Among Canadian Plastic Surgery Residents Supplemental material, sj-docx-1-psg-10.1177_22925503261477392 for Factors Associated With Research Productivity Among Canadian Plastic Surgery Residents by Colm Guyn, Justin Lee and Curtis Budden in Plastic Surgery

sj-docx-2-psg-10.1177_22925503261477392 - Supplemental material for Factors Associated With Research Productivity Among Canadian Plastic Surgery Residents Supplemental material, sj-docx-2-psg-10.1177_22925503261477392 for Factors Associated With Research Productivity Among Canadian Plastic Surgery Residents by Colm Guyn, Justin Lee and Curtis Budden in Plastic Surgery

**Figure:** Video 1.
