# Supplementary material for: Factors Associated With Research Productivity Among Canadian Plastic Surgery Residents
Source: Plast Surg (Oakv). 2026 Aug 26:22925503261477392. Online ahead of print. doi: 10.1177/22925503261477392 (PMC13570231; doi:10.1177/22925503261477392)
Supplement: Supplementary file 1 [file sj-docx-1-psg-10.1177_22925503261477392.docx]

**Supplemental Table 1.** Survey instrument: items and response options.

| **Item Number** | **Survey item** | **Response option** |
| --- | --- | --- |
| 1 | What is your age? | |
|  |  | 20–25 |
|  |  | 26–30 |
|  |  | 31–35 |
|  |  | 36–40 |
|  |  | >40 |
|  |  | Prefer not to answer |
| 2 | What is your gender? | |
|  |  | Male |
|  |  | Female |
|  |  | Other |
|  |  | Prefer not to answer |
| 3 | What is your marital status? | |
|  |  | Single |
|  |  | In a relationship |
|  |  | Living with partner |
|  |  | Married |
|  |  | Separated |
|  |  | Divorced |
|  |  | Widowed |
|  |  | Prefer not to answer |
| 4 | Do you have children? | |
|  |  | No |
|  |  | Yes |
|  |  | Prefer not to answer |
| 5 | To which residency program do you belong? | |
|  |  | Dalhousie University |
|  |  | McGill University |
|  |  | McMaster University |
|  |  | Université de Montréal |
|  |  | Université Laval |
|  |  | University of Alberta |
|  |  | University of British Columbia |
|  |  | University of Calgary |
|  |  | University of Manitoba |
|  |  | University of Ottawa |
|  |  | University of Toronto |
|  |  | Western University |
| 6 | What is your current post-graduate year of residency training? | |
|  |  | PGY1 |
|  |  | PGY2 |
|  |  | PGY3 |
|  |  | PGY4 |
|  |  | PGY5 |
|  |  | PGY 6+ |
| 7 | What Undergraduate degree did you complete? | |
|  |  | Bachelor of Science (BSc) |
|  |  | Bachelor of Health Sciences (BHSc) |
|  |  | Bachelor of Arts (BA) |
|  |  | Bachelor of Engineering (BEng) |
|  |  | Bachelor of Commerce (BCom) |
|  |  | Bachelor of Kinesiology (BKin) |
|  |  | Bachelor of Public Health (BPH) |
|  |  | Other (please specify): [Open text response] |
| 8 | Did you complete a post-graduate degree prior to medical school? | |
|  |  | No |
|  |  | Yes – Master’s degree (thesis-based) |
|  |  | Yes – Master’s degree (course-based) |
|  |  | Yes – PhD or equivalent |
|  |  | Other (please specify): [Open text response] |
| 9 | How many peer-reviewed publications did you author PRIOR to residency? | |
|  |  | [Open text response] |
| 10 | How many peer-reviewed publications have you authored currently? | |
|  |  | [Open text response] |
| 11 | If known, can you disclose your h-index (a measure of research productivity)?  If unsure it can be found at: <https://www.scopus.com/freelookup/form/author.uri>  Use your first and last name (or ORCID) to search for your profile, navigate to your profile by clicking on your name – your h-index will be included in your profile. | |
|  |  | [Open text response] |
| 12 | Did you take dedicated time off for research during/following medical school (e.g., postponed graduation)? | |
|  |  | No |
|  |  | Yes |
| 13 | Are you currently enrolled in, or have you completed, a Clinical Investigator Program (CIP) or similar thesis-based research program during residency (e.g., MSc or PhD)? | |
|  |  | No |
|  |  | Yes – Currently enrolled |
|  |  | Yes – Completed |
|  |  | Other (please specify): [Open text response] |
| 14 | Have you conducted or plan to complete a research block during residency | |
|  |  | No |
|  |  | Yes - Completed |
|  |  | Yes - Planned |
|  |  | Unsure |
|  |  | Other (please specify): [Open text response] |
| 15 | Are you interested in completing a fellowship? | |
|  |  | No |
|  |  | Yes |
|  |  | Unsure |
|  |  | Other (please specify): [Open text box] |
| 16 | Are you interested in an academic career? | |
|  |  | No |
|  |  | Yes |
|  |  | Unsure |
|  |  | Other (please specify): [Open text box] |
| 17 | On average, how many hours per week do you spend on research activities during residency? | |
|  |  | [Open text response] |
| 18 | Does your program have a mandatory research requirement for graduation? | |
|  |  | No |
|  |  | Yes |
|  |  | Unsure |
| 19 | How often does your plastic surgery division schedule meetings focused on research (e.g., journal clubs, workshops, research-in-progress sessions)? | |
|  |  | Weekly |
|  |  | Monthly |
|  |  | Quarterly |
|  |  | Less than quarterly |
|  |  | Never |
|  |  | Unsure |
| 20 | Which of the following are available to support resident research? (Select all that apply) | |
|  |  | Research director |
|  |  | Research supervisor |
|  |  | Research Assistant |
|  |  | Biostatistician |
|  |  | IT or data support |
|  |  | Internal funding |
|  |  | Other (please specify): [Open text response] |
| 21 | Have you applied for external research funding during residency | |
|  |  | No |
|  |  | Yes, it was declined |
|  |  | Yes, it was approved |

*This table lists all survey items administered to Canadian plastic surgery residents and the available response options (including open-text fields). Certain items were later categorized for analysis. Participants were allowed to submit non-responses. CIP = Clinician Investigator Program, PGY = postgraduate year.*
