# Supplementary material for: Factors Associated With Research Productivity Among Canadian Plastic Surgery Residents
Source: Plast Surg (Oakv). 2026 Aug 26:22925503261477392. Online ahead of print. doi: 10.1177/22925503261477392 (PMC13570231; doi:10.1177/22925503261477392)
Supplement: Supplementary file 2 [file sj-docx-2-psg-10.1177_22925503261477392.docx]

| **Supplemental Table 2.** Semi-structured interview outline | |
| --- | --- |
| **Domain** | **Core prompt (optional probes indented)** |
| Residency research experience | To start, could you describe your experience with research during residency so far? |
|  | - How do you feel about the amount or type of research you’ve been able to do? |
|  | - What motivates you (or demotivates you) to engage in research during residency? |
|  | - Can you share a specific example of a project that was particularly positive or challenging for you? |
| Pre-residency experiences | Can you tell me about any experiences or training prior to residency that have influenced your ability to conduct research? |
|  | - How did those experiences shape your approach to research now? |
| Program supports | What kinds of supports within your residency program have been most helpful for conducting research? |
|  | - Are there particular individuals, resources, or structures that would make a difference? |
| Program culture and expectations | How do you feel your program’s culture or expectations around research influence your research productivity? |
|  | - Can you give an example of how culture/expectations affected a project |
| Barriers and disruptions | What have been the biggest challenges to completing research during residency? |
|  | - How do time demands, scheduling, or clinical duties affect your ability to do research? |
|  | - Can you describe a time when a project stalled or did not move forward? What factors contributed? |
| Recommendations | What changes or supports do you think would most improve research involvement or success among residents? |
|  | - From what you’ve observed, any factors in particular that you feel influence some residents being more productive in research? |
|  | - What’s your perspective on how research should be approached during residency? |
| Closing | Is there anything else you’d like to add about your experience with research during residency that we haven’t discussed? |

*Interviews were semi-structured. After confirming consent, participants were informed that there were no right or wrong answers, interviews were audio-recorded, transcribed, and de-identified to preserve confidentiality. Probes were used flexibly to elicit examples and clarify responses.*
